# Attention control in a demanding dynamic time-sharing environment: An eye-tracking study

**DOI:** 10.3758/s13414-021-02377-z

**Published:** 2021-09-30

**Authors:** Jaakko Kulomäki, Lauri Oksama, Esa Rantanen, Jukka Hyönä

**Affiliations:** 1 Department of Psychology and Speech-Language Pathology, University of Turku, 20014 University of Turku, Turku, Finland; 2 Human Performance Division, Finnish Defence Research Agency, P.O. Box 5, 04401, Järvenpää, Finland; 3 Department of Psychology, Rochester Institute of Technology, Rochester, NY USA

**Keywords:** Cognitive control, Attention allocation, Time-sharing, Multitasking, Eye-tracking

## Abstract

In this study, we examined different models of cognitive control in dynamic time-sharing situations. We investigated attentional allocation by registering participants’ eye movements while they performed a new time-sharing task that forced them to solve resource conflicts between subtasks through prioritization. Participants were monitoring four subtasks each requiring different amounts of visual attention and response frequencies. Participants’ attention allocation was operationalized in terms of the time spent dwelling on subtasks, the rate they visually sampled the tasks, and the duration of dwells. Additionally, the accuracy of responses and efficiency of time-sharing were estimated. In Experiment 1, we studied adaptation to a time-sharing environment in which priority order of the subtasks was kept constant from trial to trial. We found that the participants sampled the most important subtasks more frequently, spent more time on them, and shifted their gaze earlier to them than to less important subtasks. That is, they allocated their attention according to the subtask priorities. In Experiment 2, subtask priorities changed from trial to trial. Despite the higher demands of the constantly changing situation, participants again adapted to the varying priorities of the subtasks almost instantly. Our results suggest that performance in complex and dynamic time-sharing situations is not managed by a system relying on liberal resource allocation policies and gradual learning. Instead, the participants’ rapid adaptation is more consistent with tighter executive and authoritative control and intelligent use of prioritization information.

## Introduction

Situations where several overlapping subtasks must be performed under time pressure are common in many everyday activities. For example, when drivers approach a busy intersection, they must glean potentially relevant information from several sources: other vehicles, pedestrians, and traffic signs through the windshield, monitor speed on the speedometer, and navigate the car by memorized or displayed map. These subtasks cannot be done simultaneously, as they all occupy the same, visual, channel. Therefore, drivers must focus on the most important task at any given time and allocate their attention to it at the cost of not attending to other, less important, tasks (i.e., perform subtask prioritization). Clearly, prioritization is the key to efficient and safe time-sharing performance.

As the subtasks usually differ in terms of their importance or urgency, prioritization may seem easy. It should be self-evident that drivers should not be adjusting the radio at the cost of observing vehicles and pedestrians in a busy intersection. However, sometimes people make serious errors in these situations, as less important subtasks grab their attention. Effective and safe task prioritization is paramount in many safety-critical domains such as aviation and process control. Typically, pilots, air traffic controllers, and control room operators have to simultaneously monitor a very large number of visual displays, and thus face highly demanding resource conflicts. Furthermore, the relevance of displays may vary dynamically from moment to moment. That means that the operators have to continuously monitor the priorities of the current situation and allocate their attention to the subtasks accordingly (see Bellenkes et al., 1997, for an aviation example). As situations may change very quickly, the operators have to immediately perceive and understand the changed subtask urgency and priorities, generate a new attention-allocation strategy, and allocate attention to the most important subtasks.

The present study investigated the psychology of prioritization and attention allocation during a demanding resource-conflict situation with several overlapping subtasks. For that we devised a new experimental multitask environment (see below). Our main theoretical focus was to compare different cognitive control strategies during time-sharing.

### Cognitive underpinnings of time-sharing

Theoretically, prioritization and resource-conflict resolution refer to executive functioning during time-sharing. Different cognitive architectures and managerial styles have been proposed to account for control of performance in conflict situations. These theories can be divided roughly into two schools of thought. One school proposes architectures based on strict executive control by assuming that attention is allocated to most important subtasks following directives of the central executive component or process (Baddeley, 1996; Meyer & Kieras, 1997; Norman & Shallice, 1986). The other school assumes that executive management is more liberal, allowing subtasks to negotiate and resolve resource conflicts locally without any external intervention (Salvucci & Taatgen, 2008, 2011).

According to Norman and Shallice’s (1986) classic theory, actions are represented as schemata that all have individual activation values determining when they are selected. Once selected, a schema continues operating unless it is actively switched off, until it has satisfied its goal, or until it is blocked by another more highly activated schema. Sequences of actions are represented as organized set of schemata with one, the source schema, serving as the highest order control unit. Activation of the source schema may activate other, lower level component schemas. When two or more schemas conflict in their need for resources, a scheduling mechanism called contention scheduling resolves the competition based on the activation values of the schemata. A schema with an activation value exceeding its activation threshold is selected. The activation threshold may decrease with the use of the schema.

Contention scheduling is automatic, fast, and requires no effort. In case there is no suitable schema available to satisfy the task goal (e.g., in novel or changed situations), an additional system called the Supervisory Attentional System (SAS) comes into play. The SAS can apply extra activation or inhibition to schemas in order to bias their selection by the contention scheduling mechanism. Compared to contention scheduling, the actions of the SAS are slower and require conscious effort. To work successfully, SAS utilizes a variety of information concerning environment, goals, available schemas, their aspects, and results of their past activation. The remarkable flexibility of human behavior observed in various task situations can be accounted for by the postulated supervisory system like the SAS. However, Norman and Shallice’s theory lacks a detailed description of the inner workings of the SAS, which makes it difficult to model human behavior, and has given reason for critiques to see the SAS as a cognitive homunculus.

Meyer and Kieras (1997) developed the idea of executive control further into a computational architecture called the Executive-Process Interactive Control (EPIC) to model human behavior in multitask situations. EPIC assumes the executive processes to utilize supervisory production rules. Based on EPIC, Kieras et al. (2000) have developed different models of multitasking by differentiating between an all-purpose General Executive (GE) process and specialized Customized Executive (CE) processes. GE enables operation in new, unfamiliar situations, but the process is conservative and uses strict and inflexible principles in resource allocation often resulting in poor multitask performance. CEs in turn are more liberal and use context-dependent information about tasks and their temporal relationships to enable advanced operations like resource pre-allocation, resulting in smooth multitask performance in certain contexts. Kieras et al. argued that the multitasking skill acquisition may be realized through a transition from a conservative GE to a liberal CE.

A more recent theory of human multitasking called threaded cognition (Salvucci & Taatgen, 2011) posits no distinct cognitive process or component responsible for resource allocation. Neither is prioritization nor strategizing involved. Subtasks (or threads) negotiate conflicting resource needs by themselves following simple rules. According to these rules, a subtask requests resources as soon as it needs them (“greedily”) and releases them as soon as the need is fulfilled (“politely”). When resource needs conflict with each other, the subtask with the highest urgency is served. Subtask’s urgency is determined by learning from past experience. Subtasks are continuously monitoring how their goals are achieved. If the amount of received resources has not been satisfactory to reach the goal, the subtask increases its urgency until it receives resources in adequate frequency. By adjusting the frequency of demands, a balanced allocation of resources between subtasks is achieved. The theory of threaded cognition offers a simple and elegant description of time-sharing, which also allows modeling human behavior in many contexts. So far assumptions of threaded cognition have been validated in dual-task situations such as driving and typing (e.g., Salvucci et al., 2006). However, it remains unclear how well threaded cognition accounts for more complex multitask situations involving a higher number of concurrent subtasks and rapidly changing priorities.

By assuming the existence of executive control in time-sharing, resource conflicts may be resolved through active prioritization and adaptation to situations. This resolution may be fast, flexible, and efficient, given that the executive controller (e.g., the SAS of Norman & Shallice, 1986) perceives and understands the correct priority order between subtasks and is capable of modifying the attention allocation accordingly during the task. In this case attention is allocated strictly to high-importance subtasks, whereas subtasks of less importance receive little or no resources. Executive controller’s efficiency in conflict resolution relies on its ability to utilize context-dependent information concerning environment, task goals, and past events, and on its ability to produce a proper priority-based allocation strategy. If it fails to do so, prioritization may be entirely erroneous and result in very weak time-sharing performance. Differences between individuals in their ability to multitask may therefore be attributed to differences in their executive controller’s ability to prioritize.

If there is no executive control involved in multitasking (i.e., threaded cognition), conflict resolution is realized through subtasks’ interactions with each other following simple rules and through adjusting subtask urgencies. Although this account is theoretically and computationally more elegant than models assuming higher level executive control, it may fall short of explaining fast adaptation to novel and dynamic multitasking situations. In a new situation, attention would initially be distributed evenly across all subtasks, meaning that high-priority (or urgency) tasks would be lacking resources, whereas low-priority tasks would be over-resourced. Without the ability to allocate resources strictly by utilizing external context-dependent information, adaptation happens slowly through trial and error, meaning that in dynamic situations resource allocation would be constantly lagging behind.

### Previous studies on attention allocation during time-sharing

A few studies have addressed the issue of adaptation in time-sharing situations. Janssen and Brumby (2010) studied participants’ attention allocation in a dual task of dialing while driving. They concluded that people can strategically control attention allocation to meet specific performance criteria. In a subsequent study (Janssen & Brumby, 2015), they used typing and a psychomotor tracking task to assess the effect of task difficulty and reward functions on allocation strategy. People allocated more attention to the task when it was difficult or when it matched their priorities as formalized through an incentive. Participants’ typing skills were also found to influence the choice of interleaving strategy. Wang et al. (2007, 2009) assessed payoff effects on strategy development and change in a synthetic work environment with multiple overlapping tasks (SYNWORK1 by Elsmore, 1994). They found that participants were sensitive to the payoff schedule and adopted performance strategies that reflected the relative importance of the tasks. Some studies reporting direct measures of attention allocation (i.e., eye-tracking measures) in time-sharing situations have focused on the effects of experience and expertise (e.g., Bellenkes et al., 1997; Haslbeck & Zhang, 2017; Kirby et al., 2014; Matton et al., 2016).

### Goals of the present research

Various time-sharing environments have been devised, but a significant problem with many of them is that they do not effectively tap into the essential challenge in time-sharing, namely how to allocate resources between subtasks whose resource demands are in direct conflict with each other. Tasks are often composed of subtasks that burden cognitive or sensory resources in different ways, meaning that subtasks’ mutual interference is not as strong as it could be. Also, self-paced tasks do not require participants to use their attention-controlling capacity to the maximum. To study resource conflicts directly, we devised a new monitoring task in which the subtasks have a priority order and in which effective performance requires prioritization. The task was based on the paradigm created by Rantanen (Levinthal & Rantanen, 2004; Rantanen & Levinthal, 2005). The task was designed to utilize solely the visual information channel. Task difficulty was adjusted to the level that introduced an obvious conflict in resource management. Subtasks cannot be carried out simultaneously, but successful performance requires an understanding of the priority order and the allocation of attention accordingly. The task consisted of four concurrent subtasks each with its own local goal. Subtasks’ goals contributed to the global goal of the whole task, but their impact was different. The task was inspired by the context of instrument flying, where each instrument contributes differently to the task of maintaining the control of the plane. However, our task was generic, universally applicable, and not restricted to aviation context. The subtasks were designed to be simple enough to minimize the effects of experience, skill, and domain-specific knowledge. Using this new task, we were able to manipulate subtask priorities and study the prioritization and adaptation in a time-sharing environment. We wanted to empirically examine how people solve apparent resource conflicts.

Subtask prioritization was operationalized by varying the rates of rewarded events in subtasks. For example, the event rate of a high-priority subtask was very high, thus yielding a large reward over the experimental session. That is, if participants did not rapidly react to this subtask, their accumulated score in the whole experiment was low. In contrast, in a low-priority subtask events occur relatively seldom. Thus, missing an event in such a subtask does not have much of an influence on the total performance score. Participants’ eye movements were recorded while they performed the task. Through eye-movement recordings we were able to estimate the frequency with which participants allocated their visual attention to each subtask and the time they spent visually attending each subtask.

Based on theories of visual scanning, we estimated an optimal attention allocation frequency for each subtask and compared participants’ performance to it. According to Senders (1964), effective monitoring of a dynamic display necessitates sampling of the display at a rate twice its event rate. On the other hand, Moray (1986) argued the optimal rate to be equal to the display’s event rate (see also the SEEV model of Wickens, 2015).

To examine the assumption of threaded cognition (Salvucci & Taatgen, 2011) regarding the relation between time-sharing performance and cognitive-processing speed, we measured the average dwell duration on each subtask. Shorter dwell durations are assumed to imply more efficient processing. Individual time-sharing performance was estimated with a continuous measure based on the performance scores by trials.

## Experiment 1

In this experiment, we investigated prioritization and attention-allocation schema during a demanding time-sharing situation. Participants monitored four subtasks of different event rates (and hence different priorities) while their eye movements were recorded. The event rates (priorities) for the subtasks remained constant during the whole session from trial to trial to allow participants to learn and adapt to subtasks’ individual attention demands. According to threaded cognition (Salvucci & Taatgen, 2011), attention allocation was supposed to improve from trial to trial as attention allocation policy is gradually adjusted to meet task requirements, and eventually attention should be distributed across the subtasks according to subtasks’ individual priorities. At first, attention allocation as well as time-sharing performance should be far from optimal when participants face an unfamiliar task. In fact, the threaded cognition theory predicts that at first there should no significant differences in the amount of attention allocated to each subtask. By contrast, an approach involving a tighter executive control like the SAS of Norman and Shallice (1986) would allow fast and effective adaptation to the task by assuming that task priorities are fully perceived and understood and formulated into a higher-level control strategy or schema. If task priorities are not correctly understood or a control strategy cannot be implemented, this approach predicts generally weak time-sharing performance with only slow improvement through learning in individual subtask performance.

### Method

#### Participants

Nineteen participants (psychology students of the University of Turku) were recruited for the experiment (one male, 18 females, mean age 26 years). All participants had normal or corrected-to-normal vision. Participants were asked about their active computer-gaming experience; one reported being an active gamer.

#### Apparatus

Eye movements were recorded with a desktop-mounted Eyelink1000 (SR Research Ltd., Ontario, Canada) system. Sampling frequency was 1,000 Hz. The stimuli were presented on a 21-in. CRT screen with a screen resolution of 800 × 600 pixels and a 75-Hz refresh rate. Participants were seated 57 cm from the screen, and a chin rest was used to stabilize the head. Stimuli were created with the E-prime software (Schneider et al., 2002a, 2002b).

#### Stimuli

**Fig. 1:**
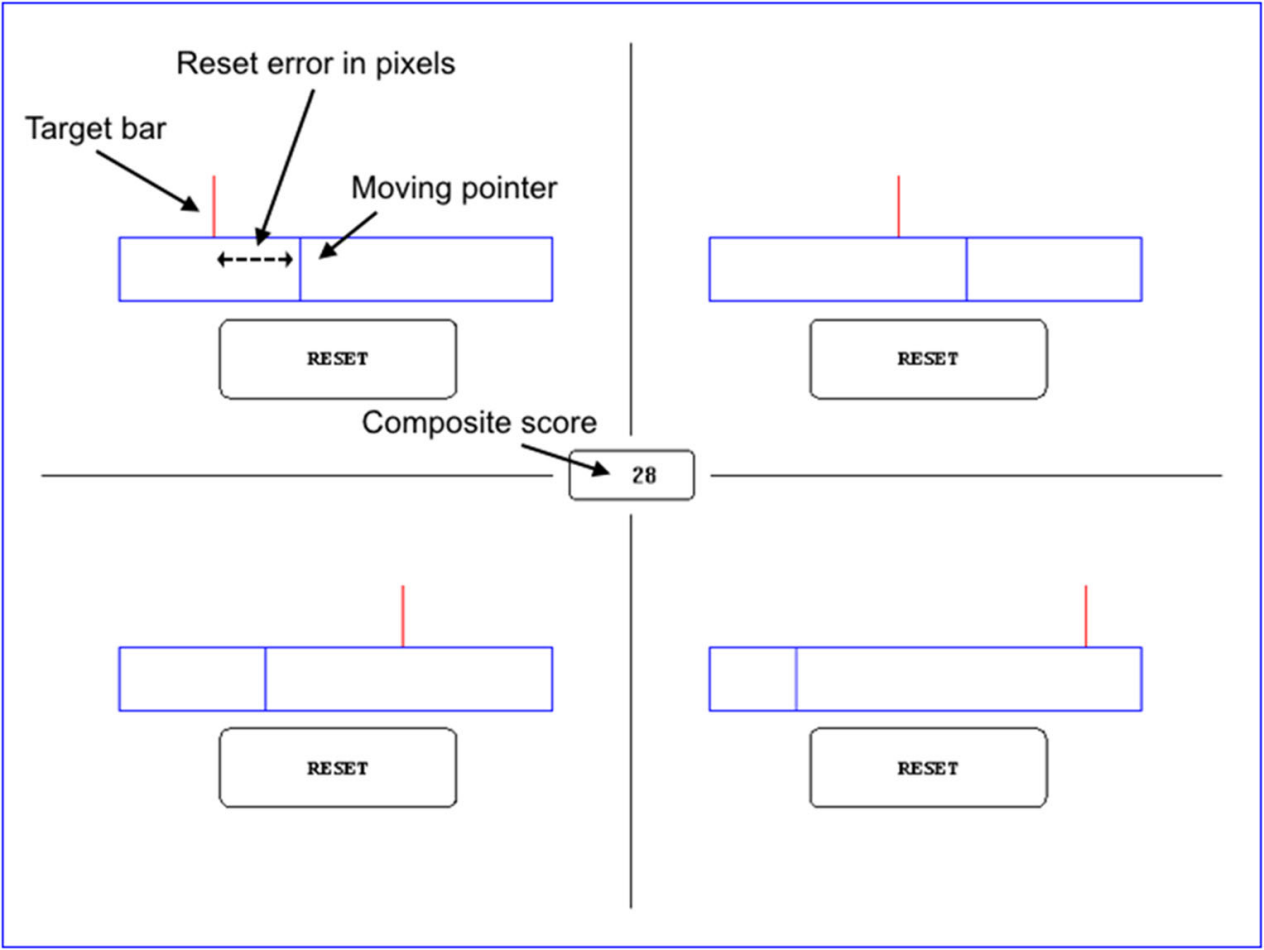
The task consisted of four indicators. Each indicator had a moving pointer and a red target bar. The pointer moved from left to right and the participant’s task was to press the reset button when pointer was aligned with the red target bar. After reset, the pointer returned to the starting position and started moving again. Accurate resets were rewarded with points. Inaccurate resets and ignoring the task were penalized with minus points. The goal of the task was to collect as many points as possible. Positioning of the target bar defined the indicator’s rate of required resets (i.e., subtask event rate) and therefore its impact on the composite score. The indicator on the upper left corner has the highest event rate and therefore the highest impact. The indicator on the lower right corner has the lowest event rate and the lowest impact. Reset error was used to measure the performance accuracy in individual subtasks. Composite score reflects the time-sharing performance level. See sample videos of the procedure and eye-movement recording: https://osf.io/5krgh/?view_only=0620319bb5f3450d977fb510e9f4c7b3

Stimuli consisted of four progress indicators, presented on a white background (see Fig. 1). The screen (29.2° VA horizontally and 21.4° VA vertically) was divided into four quarters by a black line with one indicator on each quarter. Each indicator comprised a blue frame (275 pixels/10.2° VA horizontally and 40 pixels/1.4° VA vertically), a moving blue pointer (40 pixels/1.4° VA vertically) and a stationary red target bar (40 pixels/1.4° VA vertically) above the frame. A reset button (150 pixels/5.6° VA horizontally and 50 pixels/1.8° VA vertically) was placed under each indicator. Each blue pointer moved horizontally within the frame at constant speed (20.25 pixels/0.70° VA/s) independent of each other. In the beginning of a trial, the pointer started moving automatically from the left edge of the frame and stopped once it reached the right edge of the frame. Participants were able to return the pointer to the starting position by pressing the respective reset button with the computer mouse after which the pointer started moving again. The red target bar above the frame was positioned in one of four possible positions: 60, 120, 180, or 240 pixels (2.2, 4.5, 6.7, or 8.9° VA) from the left edge of the frame. Target positions determined frequencies of pointer-target encounters (i.e., subtask event rate). The frequencies were 0.34 Hz, 0.17 Hz, 0.11 Hz, and 0.08 Hz.

#### Experimental task

Participants’ task was to monitor all the indicators and perform the task as accurately as possible by avoiding early and late resets. To motivate the participants to perform at their maximum level, a feedback system was constructed. If the reset button was pressed when the pointer was within ± 2 pixels from the target bar position (the reward zone), the participant was rewarded with 10 points signaled by a distinct reward tone. On the other hand, if the pointer was outside the reward zone at the moment of reset, the participant was penalized with -2 points signaled by a distinct penalty tone. Finally, if the pointer passed the reward zone, the participant was penalized with -2 points/s until the indicator was reset. Rewards and penalties were summed up into a composite score that was presented on the counter in the middle of the screen. Participants were instructed to pay attention to all subtasks and collect as many points as possible. No information about task priorities and allocation strategies was given to the participants.

#### Dependent variables

Eye-movement data were parsed into fixations, after which fixations were assigned to one of five areas of interest (four subtasks and score counter). Fixations shorter than 80 ms were excluded. Main dependent measures, derived from eye-movement data, were average percentage of trial time spent looking at different subtasks, visual sampling rate, and dwell time. Other dependent measures included reset error rate, time to first action, and the composite score of each trial.

#### Design

Two independent variables were manipulated: subtask event rate (0.34 Hz, 0.17 Hz, 0.11 Hz, 0.08 Hz) and amount of practice. Participants’ time-sharing performance (composite score for trials) was a factor of interest.

#### Procedure

Participants were first presented with a short Power Point presentation outlining the general procedure and explaining the trial sequence. Instructions were presented again on the computer display prior to the experiment. At the beginning of each session, the eye-tracker was calibrated using a 9-pt calibration. Drift correction was done after every trial. A chinrest was used to reduce head movements and to control the viewing distance. Participants were encouraged to practice the task for two trials after which they were given a chance to ask for further clarification. Each trial, involving all four subtasks, lasted for 1 min. There were altogether 15 trials in the session. A 15-s rest period was placed between the trials, after which the next trial started automatically. During the rest, the remaining time and the ordinal number of the next trial were displayed on the screen. After the session was completed, participants were asked to describe the performance strategy they employed (if any) during the task. Eight participants (42.1 % of all participants) reported that they focused on the subtask of highest event rate. Three participants (15.8 %) reported other strategies, while seven (36.8 %) reported having used no particular strategy. For one participant (5.3 %) this information was missing. The duration of the entire session was about 25 min.

### Results

#### Effect of practice on the composite score

**Fig. 2:**
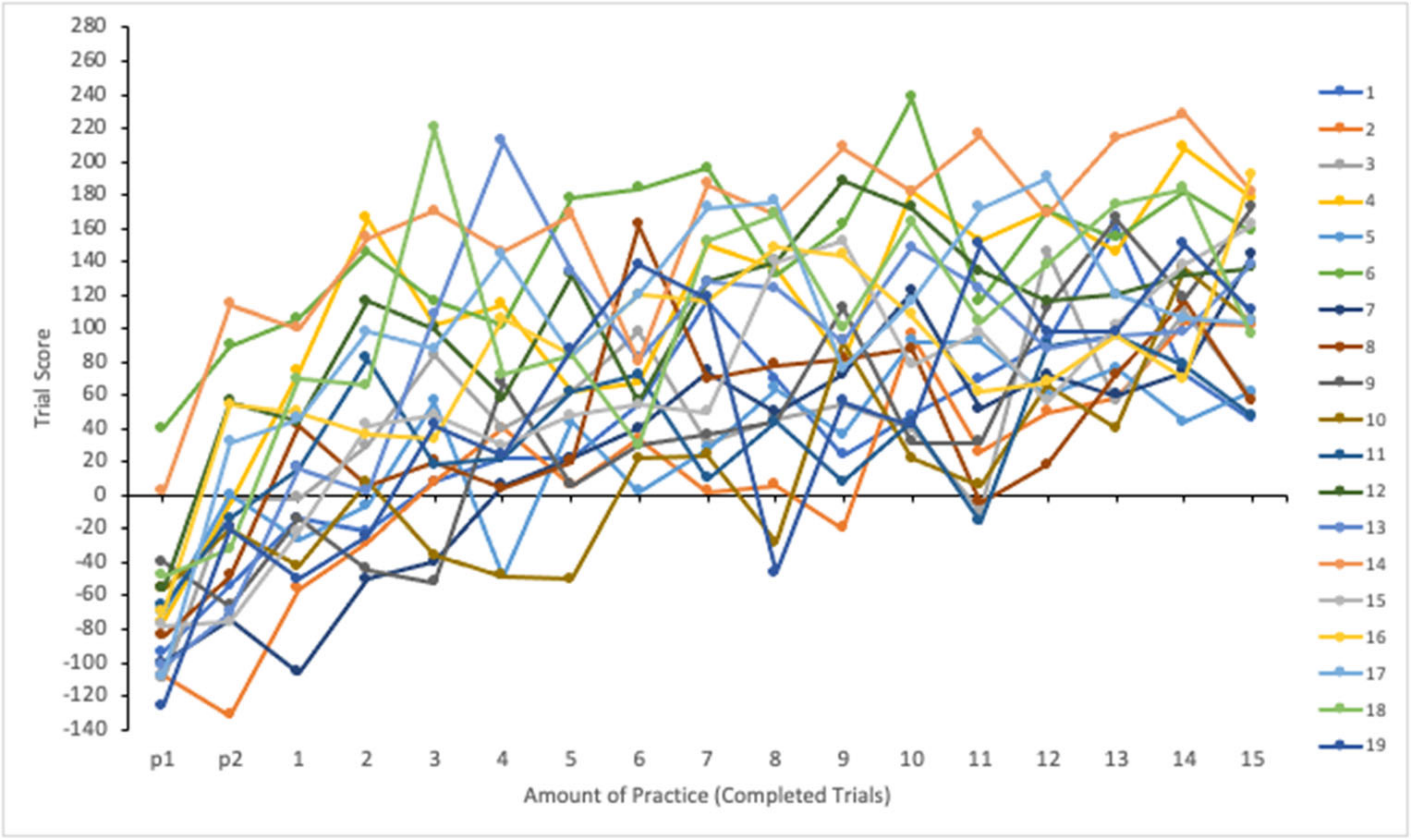
Mean scores as a function of completed trials for all participants (1–19)

We analyzed the effect of practice on participants’ task performance by calculating average scores for each trial. Figure 2 shows mean scores as a function of completed trials for all participants. Score data were submitted to a linear mixed effects analysis using SPSS 26.0.0.1 (SPSS, Inc., Chicago, IL, USA). Amount of practice (number of completed trials) was entered in the model as a fixed effect and participants as a random effect. A significant main effect of practice on average trial score was found, *F*(16, 1257.000) = 133.338, *p* < .001; participants improved their performance along the trials. As evident in Fig. 2, there is variation between participants in the level of their time-sharing performance as well as in the effect of practice. Except for two, all participants started with a negative trial score on the practice trials (p1 and p2), but then improved rather quickly. Some irregularities can be seen in participants’ level of performance from trial to trial. High trial score may be followed by a considerable drop on the next trial and vice versa.

#### Average percentage of trial time spent looking at different subtasks

**Fig. 3:**
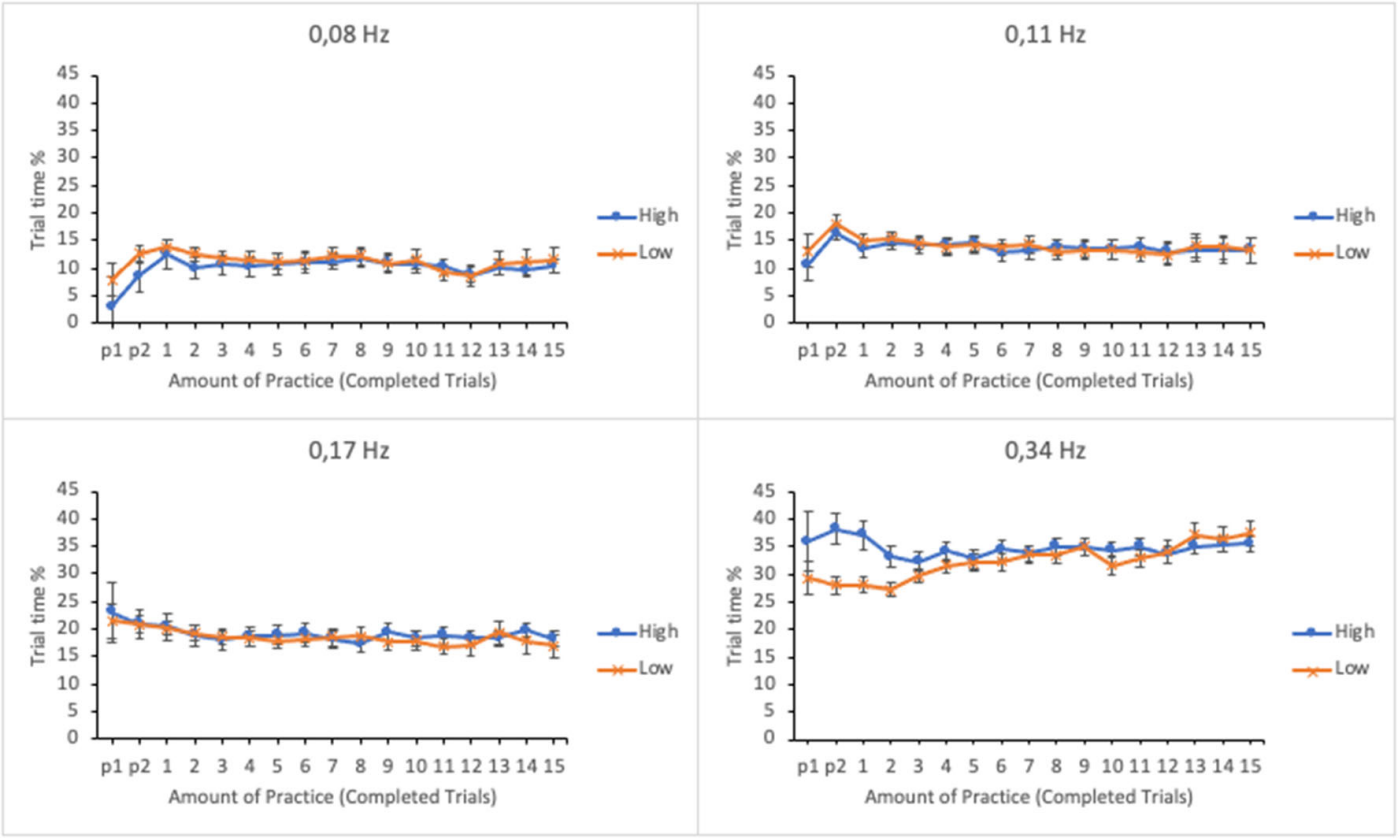
Estimated marginal means of the trial-time percentages spent in subtasks as a function of amount of practice and time-sharing performance of the lower (low-performing by the trial score) and the upper quartiles (high-performing by the trial score). The subtasks are presented in separate panes. Error bars represent the SEMs

The allocation of visual attention was analyzed by calculating the percentage of trial time participants spent fixating on the subtasks. The data were submitted to a linear mixed effects analysis. Amount of practice (number of completed trials), subtask event rate, and trial score, as well as their interactions, were entered in the model as fixed effects. Participants and its interaction with subtask event rate were entered as random effects. Results are presented in Table 1. The estimated marginal means (EMMs) of the trial time percentages as well as their standard errors as a function of practice and trial score in the four subtasks are presented in Fig. 3. To illustrate the differences between lower and higher levels of time-sharing performance, the results are plotted for the lower and the upper quartiles of the trial score distribution (36 and 132 reward points, respectively).

**Table 1:** The results of the linear mixed effects analyses for eye measures, reset accuracy, and time to first action

Effect	Percentage of trial time		Visual sampling rate		Dwell duration		Reset error		Time to first action	
	*F*	*p*	*F*	*p*	*F*	*p*	*F*	*p*	*F*	*p*
Event rate	*F*(3, 108.273) = 116.130	**<.001**	*F*(3, 128.898) = 50.986	**<.001**	*F*(3, 164.493) = 68.632	**<.001**	*F* < 1		*F*(3, 170.837) = 3.783	**.012**
Practice	*F* < 1		*F*(16, 1086.520 = 3.029	**<.001**	*F*(16, 1088.299) = 2.788	**<.001**	*F*(16, 1083.440) = 1.369	.149	*F*(16, 232.024) = 2.182	**.006**
Trial score	*F*(1, 1079.075) = 1.283	.258	*F*(1, 1087.578) = 8.082	**.005**	*F*(1, 1048.074) = 14.444	**<.001**	*F*(1, 783.325) = 19.000	**<.001**	*F* < 1	
Event rate × Practice	*F*(48, 1096.613) = 3.054	**<.001**	*F*(48, 1100.742) = 2.129	**<.001**	*F*(48, 1109.340) = 1.160	.215	*F*(48, 1094.060) = 1.181	.190	*F*(48, 777.850) = 1.141	.241
Event rate × Trial score	*F*(3, 974.850) = 6.971	**<.001**	*F*(3, 745.126) = 1.343	.259	*F*(3, 405.562) = 4.226	**.006**	*F*(3, 689.334) = 1.861	.135	*F* < 1	
Practice × Trial score	*F < 1*		*F*(16, 1085.466) = 1.689	**.043**	*F*(16, 1086.850) = 1.219	.246	*F*(16, 1081.395) = 1.872	**.019**	*F* < 1	
Event rate × Practice × Trial score	*F*(48, 1093.028) = 1.628	**.005**	*F* < 1		*F*(48, 1107.430) = 1.219	.149	*F*(48, 1090.060) = 1.473	**.021**	*F*(48, 777.518) = 1.152	.227

There was a significant main effect of subtask event rate on the percentage of trial time; the higher the event rate, the higher the percentage, indicating participants’ tendency to allocate more attention in subtasks with higher event rates. The main effects of amount of practice and trial score were not significant. The interaction between subtask event rate and trial score was significant but interaction between amount of practice and trial score was not. However, the interaction between subtask event rate and amount of practice and the three-way interaction between subtask event rate, amount of practice, and trial score were both significant.

To break apart the three-way interaction, a linear mixed effects analysis was calculated separately for each subtask by entering amount of practice and trial score and their interaction as fixed effects and participants as a random effect. It turned out that the interaction between amount of practice and trial score was only significant for the subtask with the highest (0.34 Hz) event rate, *F*(16, 271.893) = 2.467, *p* = .002 (for all the other subtasks *F*s < 1). As apparent from Fig. 3, when the subtask event rate is high, the lower-performing individuals increase the amount of visual attention along the trials. Higher-performing individuals set the level of attention initially higher and then keep it relatively unchanged throughout the session.

#### Visual sampling rate

**Fig. 4:**
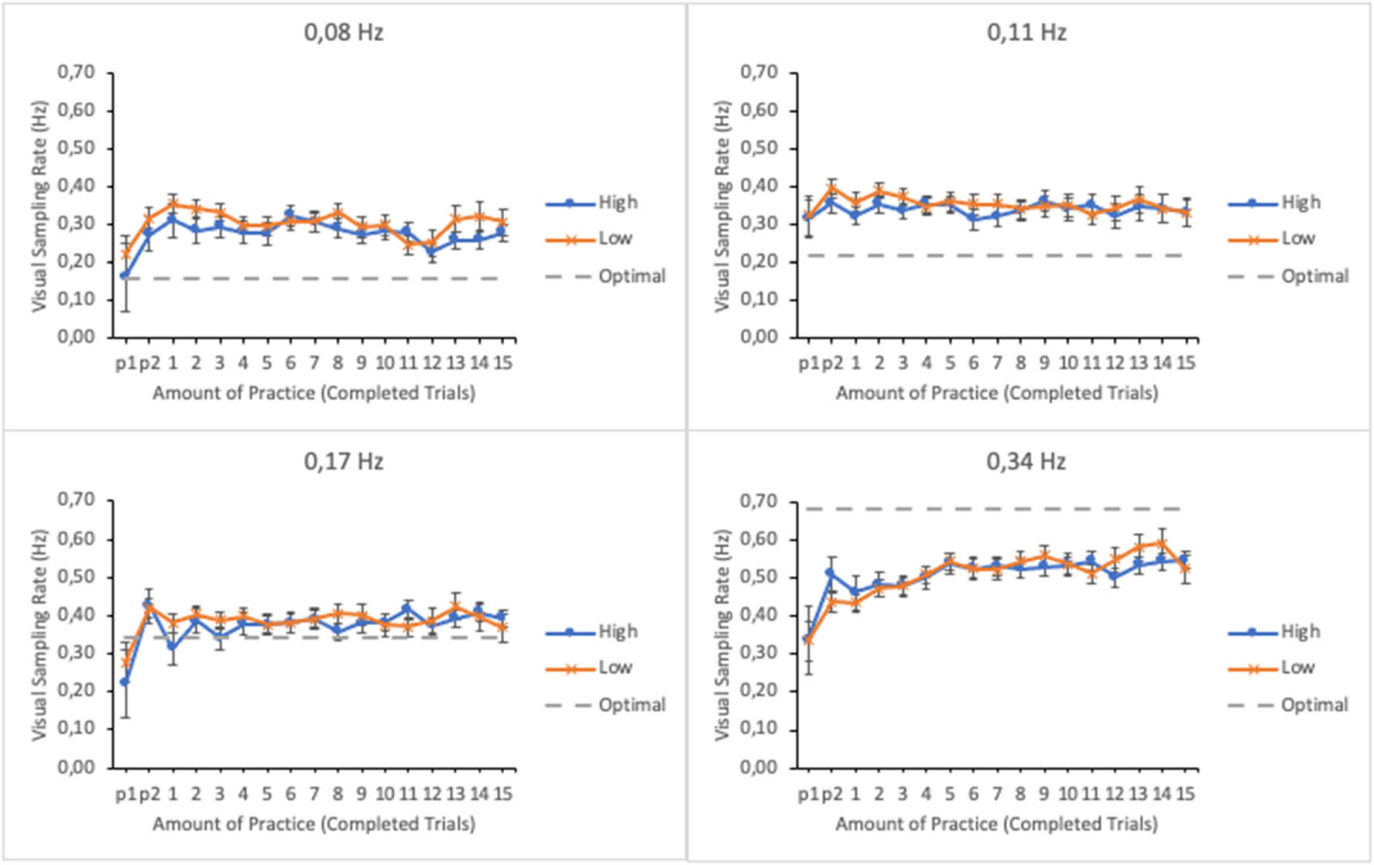
Estimated marginal means of the visual sampling rates in subtasks as a function of amount of practice and time-sharing performance of the lower (low-performing by the trial score) and the upper quartiles (high-performing by the trial score). The optimal sampling rate according to Senders (1964) is presented with dashed line. The subtasks are presented in separate panes. Error bars represent the SEMs

To further investigate attentional allocation during task performance, the rate with which the participants visually sampled the subtasks throughout the session was calculated by dividing the total dwell time spent on a subtask by the number of gaze visits (enter and leave) to it. Amount of practice, subtask event rate, and trial score, as well as their interactions, were entered in the model as fixed effects. Participants and their interaction with subtask event rate were entered as random effects. Figure 4 shows the EMMs of the visual sampling rates as well as their standard errors as a function of practice and trial score in the four subtasks.

There were significant main effects of subtask event rate, amount of practice, and trial score on the visual sampling rate (see Table 1). The interaction between subtask event rate and amount of practice as well as between amount of practice and trial score were significant. The interactions between subtask event rate and trial score and the three-way interaction between subtask event rate, amount of practice, and trial score were not significant.

Breaking apart the interactions with a linear mixed effects analysis revealed that the effect of practice was significant for the subtasks of lowest and highest event rates, *F*(16, 273.943) = 2.323, *p* = .003 and *F*(16, 272.314) = 4.461, *p* < .001, respectively. Interaction terms for the other subtasks were non-significant: *F*(16, 272.976) = 1.253, *p* = .228 (0.11 Hz) and *F* < 1 (0.17 Hz). There was increase in visual sampling rate of the subtask of highest event rate along the trials and a slight decrease for the subtask of lowest event rate, whereas the sampling rate remained steady for the other subtasks (see Fig. 4).

The optimal sampling rates suggested by Senders (1964) (two times the task event rate) are plotted in Fig. 4 for each subtask. It can be seen that participants were sampling the subtasks of event rates 0.08 Hz and 0.11 Hz excessively. For the subtask of 0.17 Hz event rate, the sampling was quite close to the optimal, and for the subtask of highest event rate the sampling rate was too low. Thus, the aforementioned changes in sampling rates along the trials for the subtasks of 0.08 Hz and 0.34 Hz reflect participants’ attempts to adjust their sampling behavior to subtasks’ requirements. Notice, however, that according to the optimal sampling rates suggested by Moray (1986) and Wickens (2015) (equal to display’s event rate) the observed sampling rates would indicate excessive oversampling on every subtask. In this case Senders’ estimate seem to fit the data better.

#### Mean dwell duration

**Fig. 5:**
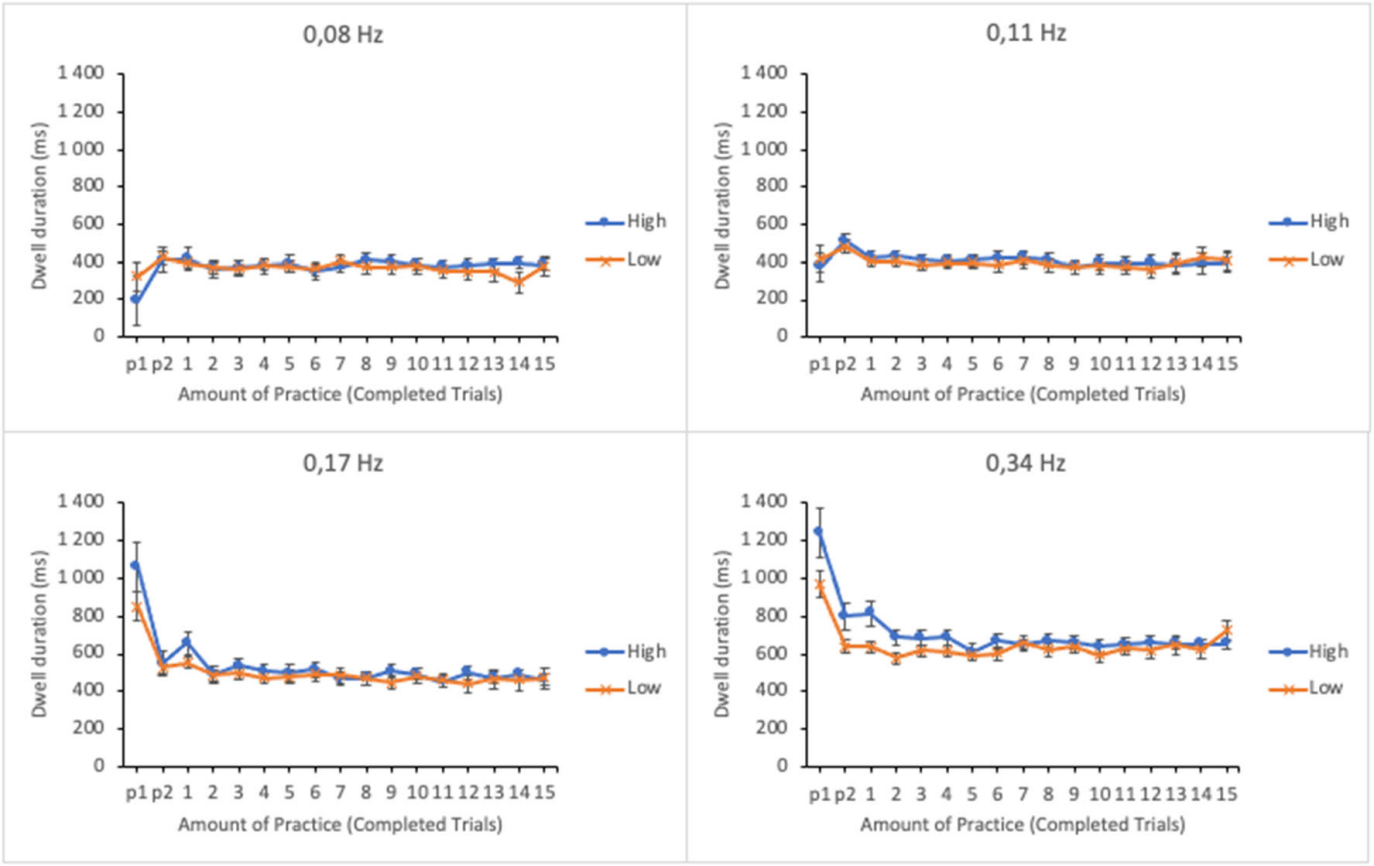
Estimated marginal means of dwell durations in the subtasks as a function of amount of practice and time-sharing performance of the lower (low-performing by the trial score) and the upper quartiles (high-performing by the trial score). The subtasks are presented in separate panes. Error bars represent the SEMs

The efficiency of information acquisition was analyzed by calculating the mean dwell duration for each subtask. Short duration is assumed to reflect high efficiency. Duration data were submitted to a linear mixed effects analysis. Amount of practice, subtask event rate, and trial score as well as their interactions were entered in the model as fixed effects. Participants and its interaction with subtask event rate were entered as random effects. The dwell durations as well as their standard errors as a function of amount of practice and trial score (at 1st and 3rd quartiles of trial score distribution) in four subtasks are shown in Fig. 5.

The main effect of subtask event rate on average dwell duration was significant (see Table 1); dwells were longer for the subtasks with higher event rates. Analogously to the trial time percentage and the frequency of visual sampling, participants seem to differentiate between the subtasks with dwell durations as well. The main effects of amount of practice and trial score were also significant. As is evident from Fig. 5, the dwell durations during the first few trials differed considerably from the durations of the later trials on all subtasks. For subtasks of higher event rates (0.34 Hz and 0.17 Hz), the dwell durations were almost twice as long during the first practice trial than on the rest of the trials. This suggests that participants initially examined these two subtasks more intensely. The two-way interaction between subtask event rate and trial score was significant. No other significant interactions were found.

The interaction was broken apart by calculating a linear mixed effects analysis separately for each subtask by entering amount of practice and trial score and their interaction as fixed effects and participants as a random effect. The observed effect of trial score was significant for the subtask of 0.34 Hz event rate only, *F*(1, 271.970) = 10.897, *p* = .001. The interaction terms for other subtasks were non-significant: *F* < 1 (0.08 Hz), *F*(1, 281.309) = 1.566, *p* = .212 (0.11 Hz) and *F*(1, 261.006) = 3.743, *p* = .054 (0.17 Hz). As is evident from Fig. 5, high performers’ dwell durations were longer on subtasks with highest event rate than those of low performers for the first third of the session. This may reflect high performers’ stronger effort to adapt to the features of the most important subtask.

#### Reset accuracy

**Fig. 6:**
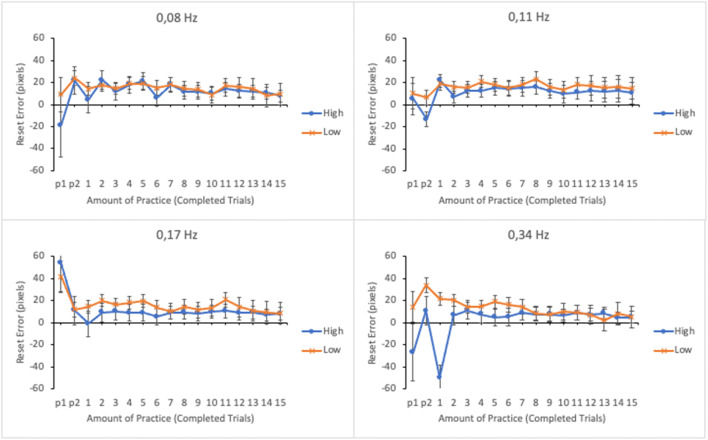
Estimated marginal means of absolute reset error in the subtasks as a function of amount practice and time-sharing performance of the lower (low-performing by the trial score) and the upper quartiles (high-performing by the trial score). The subtasks are presented in separate panes. Error bars represent the SEMs

The level of performance for each subtask was analyzed by computing the accuracy of resets. We calculated the mean absolute reset error in pixels for each subtask in each trial. Small error in resets reflects high accuracy. Error data were submitted to a linear mixed effects analysis. Amount of practice, subtask event rate, and trial score as well as their interactions were entered as fixed effects in the model. Participants and its interaction with subtask event rate were entered as random effects. For illustrative purposes the EMMs of reset error as well as their standard errors as a function of amount practice and trial score (the lower and the upper quartiles of trial score distribution) in the four subtasks are shown in Fig. 6.

The main effects of subtask event rate and amount of practice on reset accuracy were not significant (see Table 1). On the other hand, the main effect of trial score was significant. The interactions between subtask event rate and amount of practice and between subtask event rate and trial score were not significant. However, there were significant interactions between amount of practice and trial score and between subtask event rate, amount of practice, and trial score.

To break apart the three-way interaction, a linear mixed effects analysis was calculated separately for each subtask by entering amount of practice and trial score and their interactions as fixed effects and participants as a random effect. The interaction between amount of practice and trial score was significant only for the subtask with the highest (0.34 Hz) event rate, *F*(16, 271.944) = 2.235, *p* = .005. The interaction terms for the other subtasks were as follows: 0.17 Hz: *F*(16, 270.159) = 1.664, *p* = .053; 0.11 Hz: *F* < 1; 0.08 Hz: *F* < 1. As can be seen in Fig. 6, reset accuracy in the subtask with highest event rate was initially poor among the lower-performing participants but improved with practice. On the other hand, the higher-performing participants achieved a higher level of accuracy, which remained relatively stable throughout the trials. Note that the negative reset errors in the first trials are due to the extrapolation when calculating the EMMs based on the model as the original reset error values were all positive.

#### Time to first action (TFA)

**Fig. 7:**
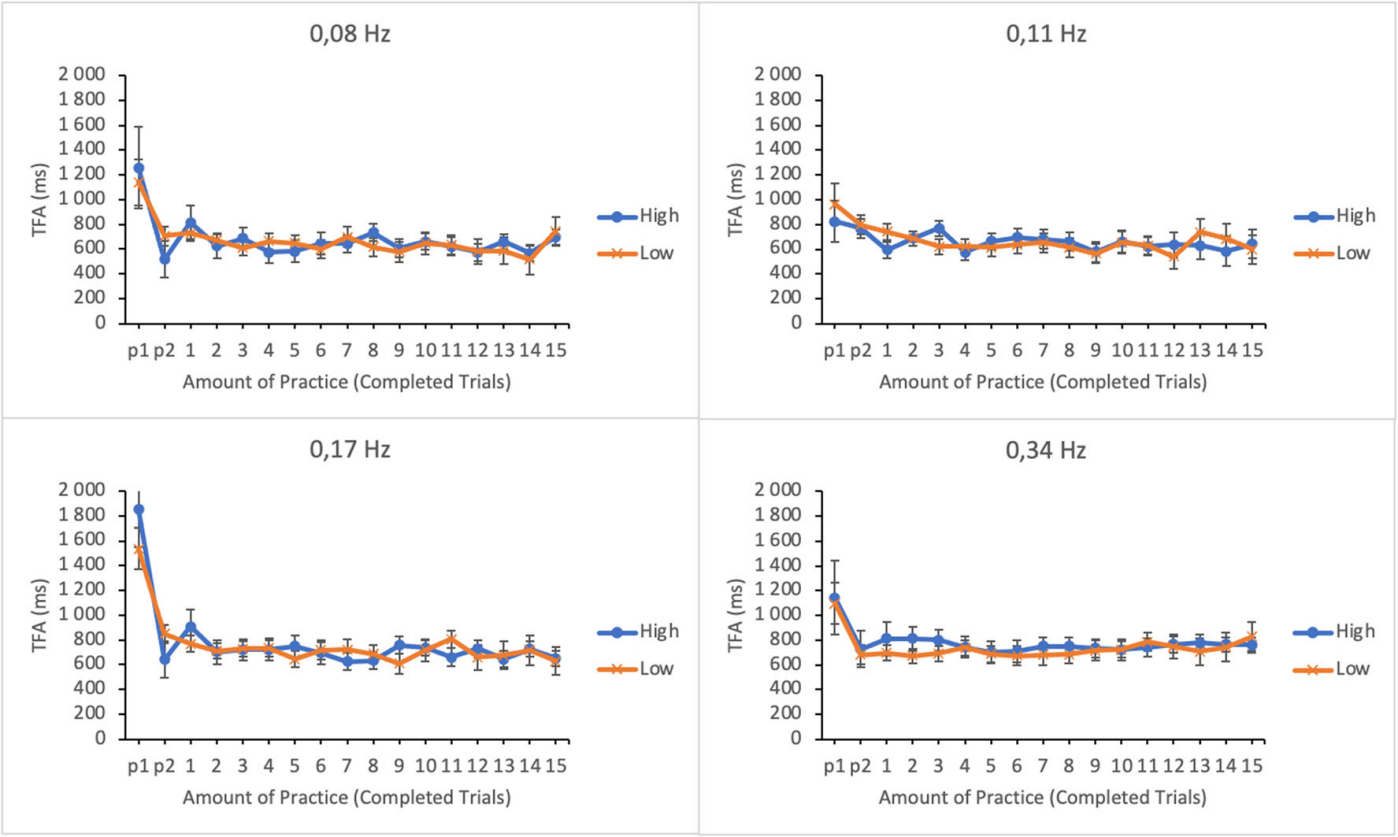
Estimated marginal means of time to first action in the subtasks as a function of amount practice and time-sharing performance of the lower (low-performing by the trial score) and the upper quartiles (high-performing by the trial score). The subtasks are presented in separate panes. Error bars represent the SEMs

The efficiency of time-sharing was analyzed by calculating the average time to first action on each subtask (following the idea presented in Rantanen, 2009). TFA was determined by calculating the time between the moment participants’ gaze entered a subtask and the moment of the following reset action (from the onset of dwell to response). Shorter TFAs were assumed to indicate more precise attention shifting and thus more efficient time-sharing. TFA data were submitted to a linear mixed effects analysis. Amount of practice, subtask event rate, and trial score as well as their interactions were entered as fixed effects in the model. Participants and its interactions with subtask event rate and amount of practice were entered as random effects. For illustrative purposes the EMMs of TFA as well as their standard errors as a function of amount practice and trial score (the lower and the upper quartiles of trial score distribution) in the four subtasks are shown in Fig. 7.

There were significant main effects of subtask event rate and amount of practice on time to first action (see Table 1). The main effect of trial score was not significant. No significant two-way interactions were found between any of the variables. The TFAs were generally longer on subtasks with higher event rates. TFAs were also longer during the first few trials but they rapidly dropped to a relatively constant level for the rest of the session.

### Discussion

The results of Experiment 1 suggest that all participants were able recognize subtasks’ different attentional requirements rapidly and allocated their attention accordingly. Participants invested more attention in terms of percentage of trial time, visual sampling rate, dwell duration, and time to first action to subtasks of higher event rates than subtasks of lower event rates. This distinction was apparent from the very first trials of the session. This clearly indicates that they were prioritizing the subtasks.

In terms of the rate of visual sampling, the amount of allocated attention was excessive in all subtasks except for the one with highest event rate in which it was insufficient compared to the optimal. However, participants increased the rate on this subtask along the trials, which suggests that participants recognized this particular subtask as the most important and strived to optimize their performance in it. Also, participants’ reporting of their strategies during the task indicate that almost half of the participants consciously recognized the priority of this subtask. The analysis of times to first action revealed that the shifting of attention preceding the reset took place earlier on subtasks of higher event rates than on the subtasks of lower event rates. This also supports the idea of prioritization. Obviously, participants wanted to make sure that they didn’t miss the moment of the reset action on the subtasks they perceived as most important by executing the attention shift early enough.

Practice didn’t have a substantial effect on participants’ attention allocation. Participants determined the level of attention to each subtask almost instantly and made only minor adjustments during the trials. The effect of practice was observed most clearly on the subtask of highest event rate in which all participants increased the visual sampling rate during the session. Also, the lower performing participants increased the percentage of trial time for the subtask of highest event rate along the trials.

The results from dwell time duration analysis do not support the prediction that higher performers’ superior performance is due to their ability to process information faster than done by low performers. The analyses showed that higher performing participants’ dwell durations were actually longer than lower performers’ on the subtask of highest event rate during the first part of the session. This suggests that information acquisition speed is not a crucial factor behind efficient time-sharing. Longer dwells may indicate that higher performers analyzed the most important subtask more intensively at first to create an accurate mental model of the required actions. This may also reflect their capability to resist premature attention shifts enabling them to absorb more information about the subtask status, thus allowing for more accurate performance control, which then resulted in more accurate resets.

Participants’ behavior is mostly inconsistent with the predictions drawn from the assumptions of threaded cognition. All participants established almost instantly a clear distinction between subtasks in regard to attention allocation, which is not in line with a gradual optimization proposed by threaded cognition. Although such behavior was observed to some extent during the first few trials, the amount of allocated attention was clearly distinctive on each subtask from the very beginning.

## Experiment 2

In Experiment 2, we put participants’ attention allocation and priority perception into a more demanding resource conflict test by introducing continuous trial-to-trial changes in subtask attention requirements. Priority changes were designed to resemble events encountered in real life (e.g., traffic), where subtask priorities may change continuously and quickly. In Experiment 1, participants were allowed to adapt their attention allocation to a single set of subtask requirements throughout the entire experimental session, whereas in Experiment 2 the set was changed after each trial so that participants were thus forced to reformulate their allocation strategy from trial to trial.

According to the theory of threaded cognition (Salvucci & Taatgen, 2011), fast prioritization changes should be very difficult for the attentional control system. Continuous priority changes should make gradual optimization considerably difficult if not impossible, and performance should remain equally poor across the trials. Due to constant changes in priorities, sampling frequencies learned from one trial cannot be utilized in the next trial, and participants are forced to start the adaptation from scratch (or even worse, if there is negative transfer/inhibition between trials).

By contrast, an attentional control system guided by a tight but agile executive could perform better in this situation. As it does not have to negotiate with its subordinates, it is able to allocate attention flexibly to meet the requirements of each subtask. If the system for some reason is unable to recognize subtask priorities correctly, performance would be very poor throughout the trials.

### Method

#### Participants

Eighteen participants (psychology students of the University of Turku, different from those in Experiment 1) were recruited for the experiment (two males, 16 females, mean age 25 years). All participants had normal or corrected-to-normal vision. Three participants reported themselves as being active gamers.

#### Apparatus, stimuli, procedure, and design

Apparatus was the same as that used in Experiment 1. The same stimuli were used as in Experiment 1, but the locations of subtasks on the screen were randomized for each trial. Participants’ task was the same as in Experiment 1, as were the experimental procedure and design.

Eight participants (44.4 % of all participants) reported that they focused on the subtask of highest event rate. Four participants (22.2 %) reported other strategies, while four (22.2 %) reported having used no particular strategy. This information was missing for two participants (11.2 %).

#### Eye-movement analysis

The analysis procedure was identical to that of Experiment 1. Due to the randomization of subtasks’ locations, subtasks were assigned to areas of interest separately for each trial.

### Results

#### Effect of practice on the composite score

**Fig. 8:**
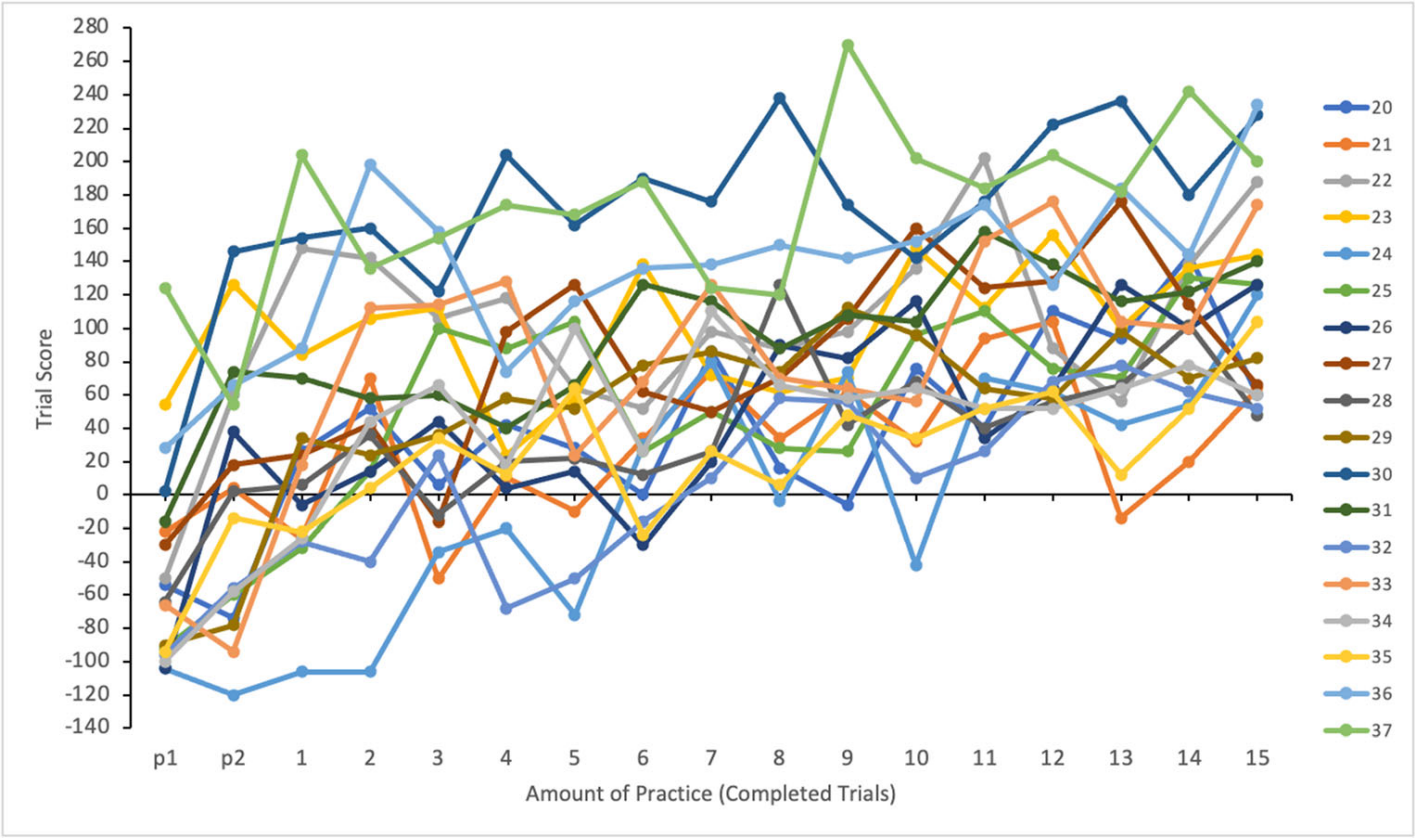
Mean scores as a function of completed trials for all participants (20–37)

We analyzed the effect of practice on participants’ task performance by calculating average scores for each trial. Figure 8 shows mean scores as a function of completed trials for all participants. Score data were submitted to a linear mixed effects analysis. Amount of practice (number of completed trials) was entered in the model as a fixed effect and participants as a random effect. Results were similar to Experiment 1. The main effect of practice on average trial score was significant, *F*(16, 1190.000) = 96.519, *p* < .001; participants improved their performance along the trials. Significant individual variability was also observed in the level of time-sharing performance as well as in the effect of practice.

#### Average percentage of trial time spent looking at different subtasks

**Fig. 9:**
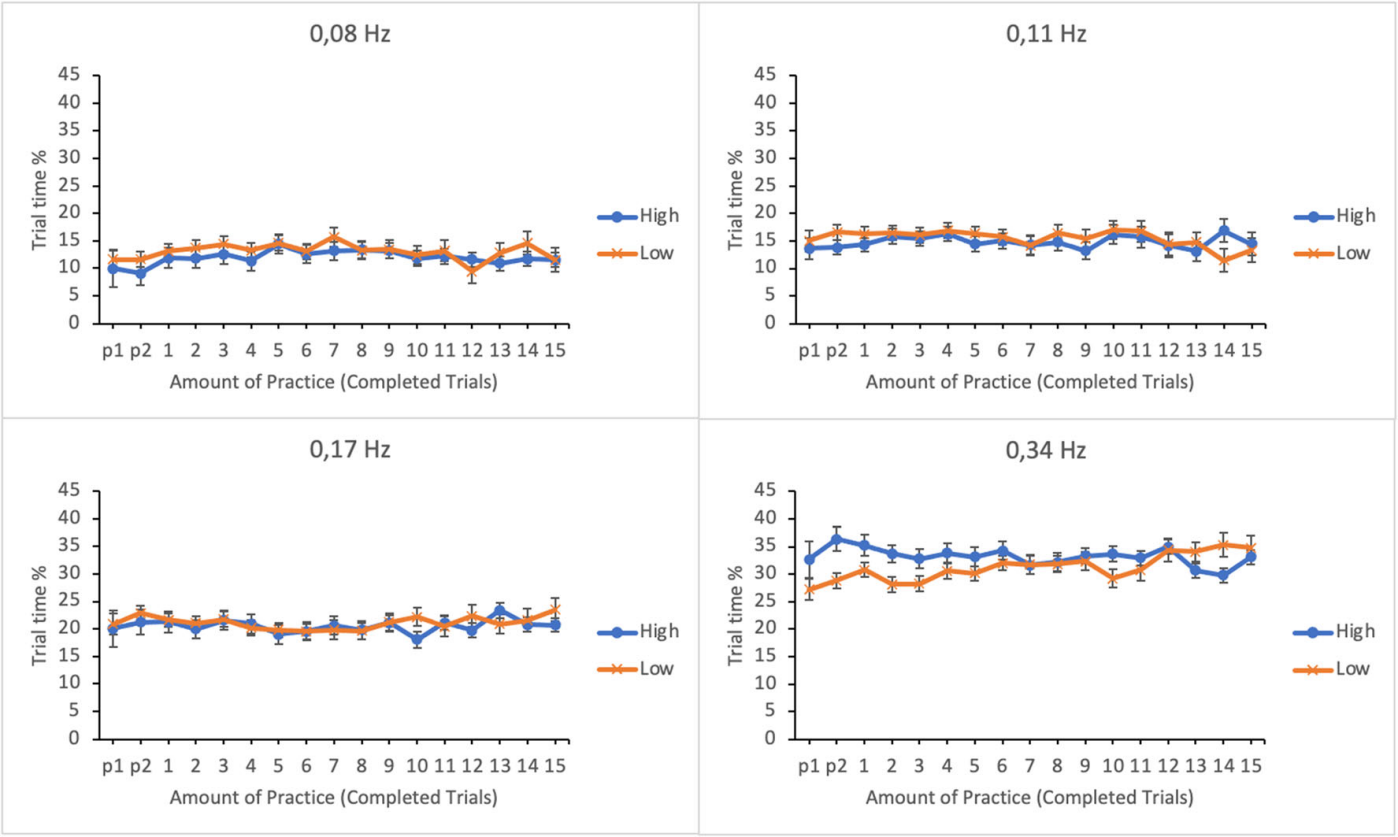
Estimated marginal means of the trial time percentage in the subtasks as a function of amount of practice and time-sharing performance of the lower (low-performing by the trial score) and the upper quartiles (high-performing by the trial score). The subtasks are presented in separate panes. Error bars represent the SEMs

Percentages of trial time spent dwelling on subtasks were submitted to a linear mixed effects analysis. Amount of practice, subtask event rate, and trial score as well as their interactions were entered in the model as fixed effects. Participants and its interaction with subtask event rate were entered as random effects. The results are presented in Table 2. Figure 9 shows the EMMs of the trial time percentages as well as their standard errors as a function of amount practice and trial score in the four subtasks. To illustrate the differences between low and high levels of time-sharing performance, the results are plotted separately for the lower and the higher quartiles of the trial composite score distribution (34 and 124 reward points respectively).

**Table 2:** The results of the linear mixed effects analyses for eye measures, reset accuracy, and time to first action

Effect	Percentage of trial time		Visual sampling rate		Dwell duration		Reset error		Time to first action	
	*F*	*p*	*F*	*p*	*F*	*p*	*F*	*p*	*F*	*p*
Event rate	*F*(3, 97.324) = 73.845	**<.001**	*F*(3, 106.238) = 15.296	**<.001**	*F*(3, 110.073) = 81.122	**<.001**	*F(*3, 100.721) = 1.359	.260	*F*(3, 105.618) = 2.905	**.038**
Practice	*F* < 1		*F*(16, 1025.992) = 2.298	**.003**	*F*(16, 1024.563) = 1.271	.208	*F(*16, 998.502) = 1.243	.228	*F(*16, 240.837) = 1.160	.302
Trial score	*F* < 1		*F(*1, 902.177) = 6.804	**.009**	*F(*1, 958.929) = 2.589	.108	*F*(1, 344.133) = 30.702	**<.001**	*F(*1, 224.670) = 1.967	.162
Event rate x Practice	*F*(48, 1034.862) = 1.742	**.002**	*F* < 1		*F*(48, 1041.306) = 1.128	.258	*F*(48, 1014.100) = 1.374	**.048**	*F*(48, 759.512) = 1.087	.321
Event rate x Trial score	*F(*3, 648.025) = 5.423	**.001**	*F*(3, 467.208) = 1.456	.226	*F*(3, 297.490) = 3.284	**.021**	*F*(3, 205.928) = 3.009	**.031**	*F(*3, 180.128) = 1.037	.378
Practice x Trial score	*F < 1*		*F* < 1		*F < 1*		*F*(16, 993.763) = 1.769	**.031**	*F*(16, 237.840) = 1.061	.394
Event rate x Practice x Trial score	*F(*48, 1027.592) = 2.169	**<.001**	*F* < 1		*F*(48, 1034.352) = 1.744	**.002**	*F*(48, 1005.965) = 1.728	**.002**	*F*(48, 754.037) = 1.122	.268

As in Experiment 1, there was a significant main effect of subtask event rate in participants’ trial time percentage; subtasks with higher event rates received more visual attention than subtasks with lower event rates. The main effects of amount practice and trial score were not significant.

The two-way interaction between subtask event rate and amount of practice was significant and so was the interaction between subtask event rate and trial score. Moreover, a significant three-way interaction was found between subtask event rate, amount of practice and trial score.

The interaction was broken apart by calculating a linear mixed effects analysis separately for each subtask by entering amount of practice, trial score and their interactions as fixed effects and participants as a random effect. As in Experiment 1, the interaction was only significant for the subtask of 0.34 Hz event rate, *F*(16, 256.314) = 2.916, *p* < .001. The interaction terms for the other subtasks were as follows: 0.17 Hz: *F* < 1; 0.11 Hz: *F*(16, 256.938) = 1.227, *p* = .247; 0.08 Hz: *F* < 1. The lower-performing participants initially assigned less trial time to the subtask of highest event rate but increased it during the subsequent trials, whereas the high-performing participants maintained a constant level of attention throughout the trials (see Fig. 9).

#### Visual sampling rate

**Fig. 10:**
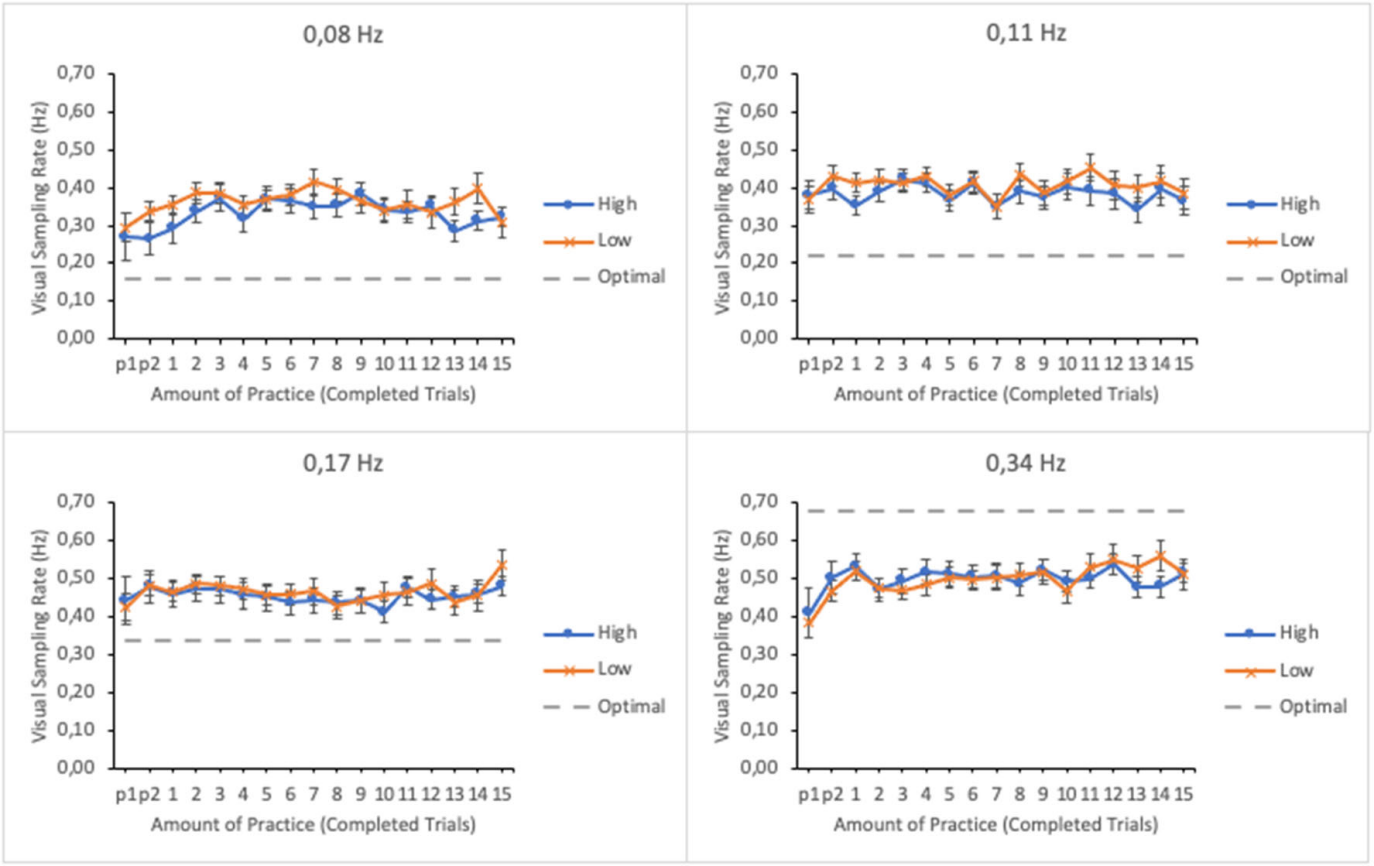
Estimated marginal means of the visual sampling rate in the subtasks as a function of amount of practice and time-sharing performance of the lower (low-performing by the trial score) and the upper quartiles (high-performing by the trial score). The optimal sampling rate according to Senders (1964) is presented with dashed line. The subtasks are presented in separate panes. Error bars represent the SEMs

Visual sampling rates were submitted to a linear mixed effects analysis. Amount of practice, subtask event rate, and trial score as well as their interactions were entered in the model as fixed effects. Participants and its interaction with subtask event rate were entered as random effects. Figure 10 shows the EMMs of the visual sampling rates as well as their standard errors as a function of amount practice and trial score in the four subtasks.

As in Experiment 1, there was a significant main effect of subtask event rate in participants’ visual sampling rate (see Table 2); subtasks with higher event rates were sampled more frequently that subtasks with lower event rates. The main effects of amount practice and trial score were also significant. No significant interactions were found.

Compared to the optimal sampling rate, participants oversampled all subtasks but the one with highest event rate. The oversampling was more obvious than in Experiment 1. As in Experiment 1, the participants undersampled the subtask of highest event rate but this time no increase in sampling rate during the session was observed.

#### Mean dwell duration

**Fig. 11:**
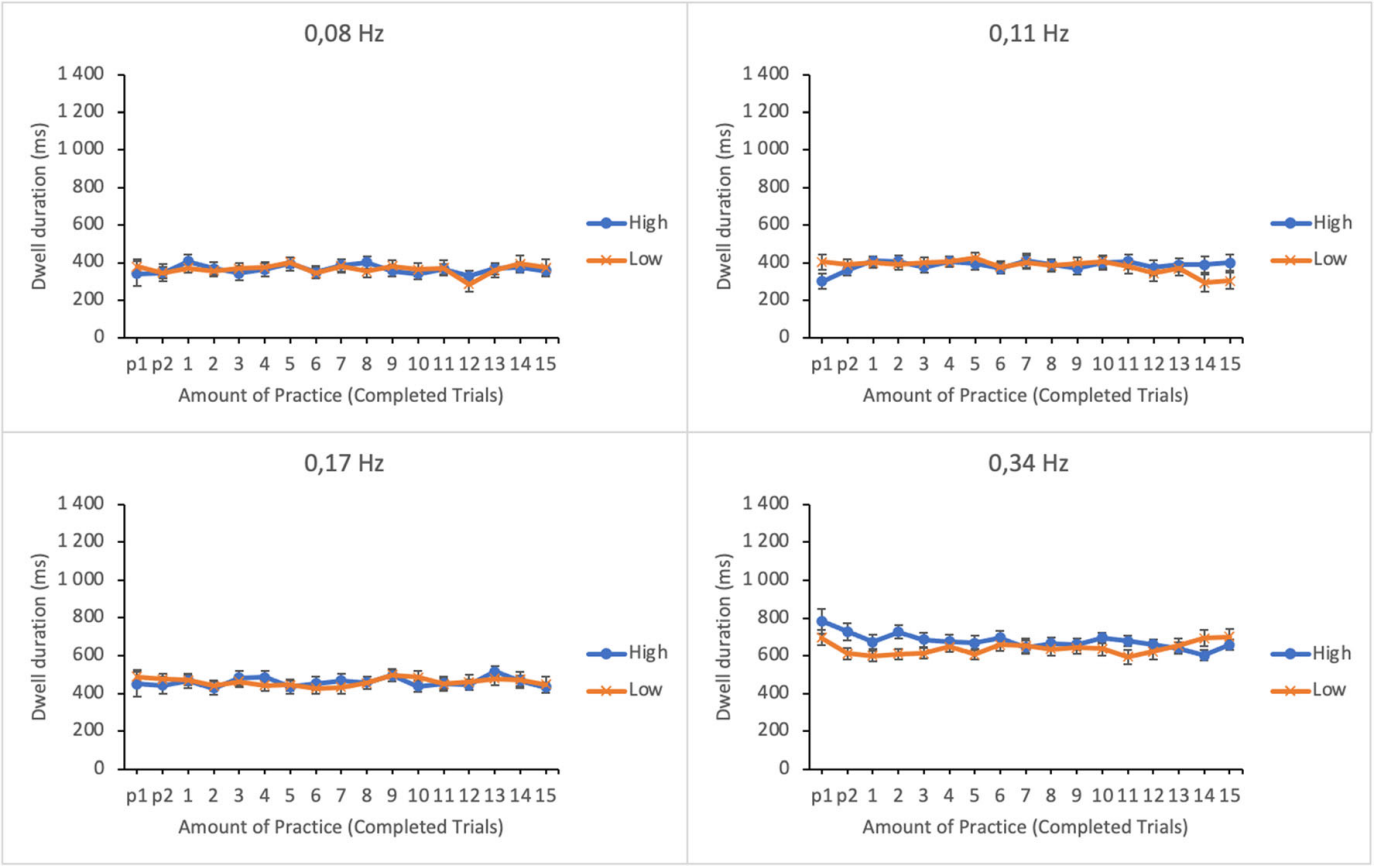
Estimated marginal means of dwell durations in the subtasks as a function of amount of practice and time-sharing performance of the lower (low-performing by the trial score) and the upper quartiles (high-performing by the trial score). The subtasks are presented in separate panes. Error bars represent the SEMs

Duration data were submitted to a linear mixed effects analysis using amount of practice, subtask event rate, and trial score as well as their interactions as fixed effects and participants and its interaction with subtask event rate as random effects. Dwell durations as a function of amount of practice and time-sharing performance are shown in Fig. 11. The main effect of subtask event rate on average dwell duration was significant (see Table 2); dwells were longer on subtasks with higher event rates. The main effects of amount of practice and trial score were not significant.

The two-way interactions between subtask event rate and amount of practice as well as amount of practice and trial score were non-significant. However, the interaction between subtask event rate and trial score and the three-way interaction between subtask event rate, amount of practice, and trial score were significant. The three-way interaction was broken apart by calculating a linear mixed effects analysis separately for each subtask by entering amount of practice and trial score and their interactions as fixed effects and participants as a random effect. The interaction was only significant for subtask with 0.11 Hz event rate, *F*(16, 256.136) = 2.815, *p* < .001. The interaction terms for the other subtasks were non-significant: 0.34 Hz: *F*(16, 256.247) = 1.527, *p* = .090; 0.17 Hz: *F* < 1; 0.08 Hz: *F* < 1. Although the interaction only approached significance for the subtask with the highest event rate, it is noticeable that, similar to Experiment 1, the higher-performing participants devoted longer dwells on the task than the lower-performing participants at the beginning of the session.

#### Reset accuracy

**Fig. 12:**
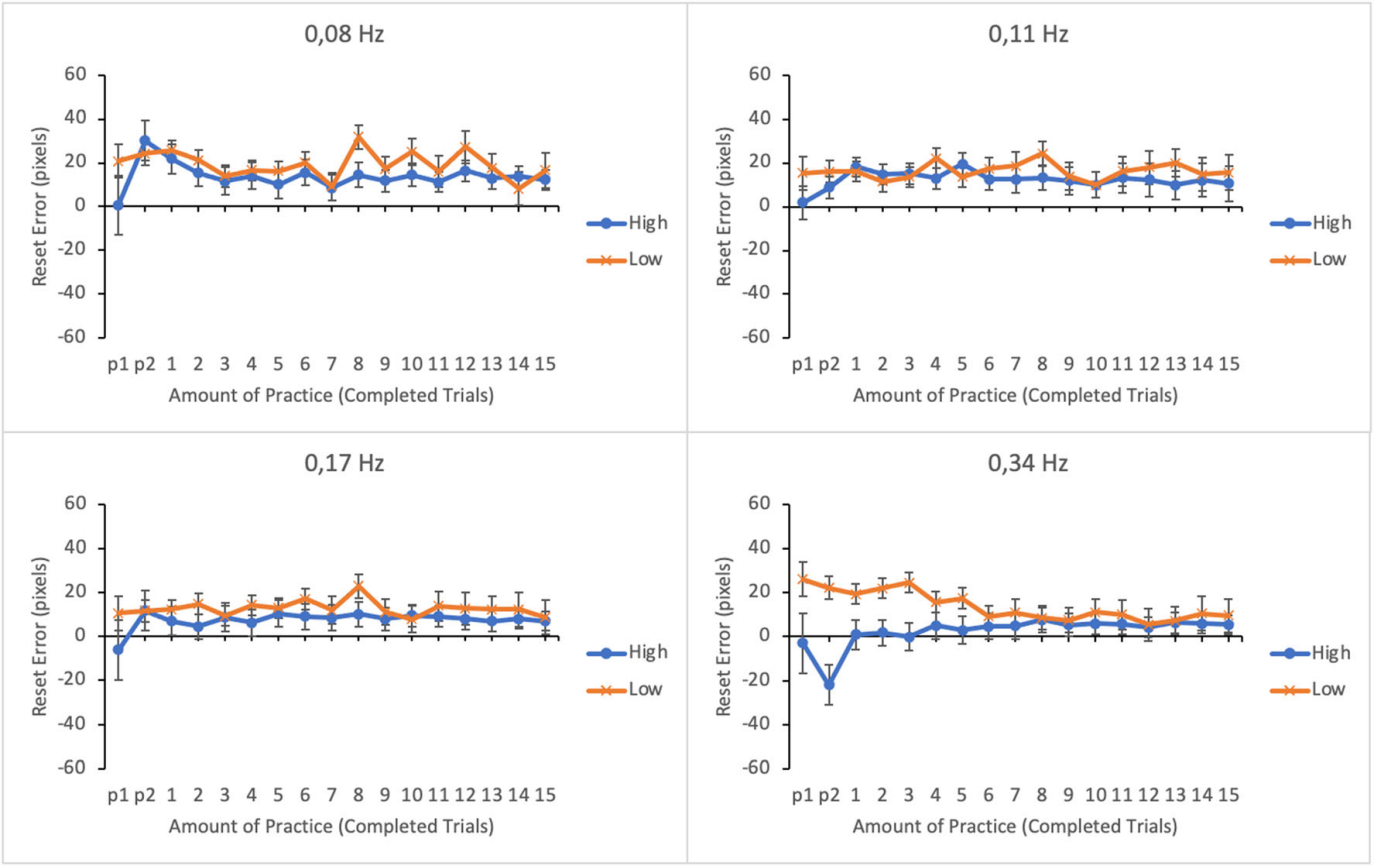
Estimated marginal means of the absolute reset error in the subtasks as a function of amount of practice and time-sharing performance of the lower (low-performing by the trial score) and the upper quartiles (high-performing by the trial score). The subtasks are presented in separate panes. Error bars represent the SEMs

The main effects of subtask event rate and amount of practice on reset accuracy were not significant (see Table 2). However, a significant main effect was found for trial score. The interactions between subtask event rate and trial score, between amount of practice and trial score and between subtask event rate and amount of practice were all significant. The three-way interaction between subtask event rate, amount of practice, and trial score was significant as well. The three-way interaction was broken apart by calculating a linear mixed effects analysis separately for each subtask by entering amount of practice and trial score and their interactions as fixed effects and participants as a random effect. A significant interaction was found for the subtask with 0.34 Hz event rate, *F*(16, 254.553) = 3.659, *p* < .001. The interaction terms for the other subtasks were non-significant: 0.17 Hz: *F*(16, 257.328) = 1.099, *p* = .356; 0.11 Hz: *F*(16, 243.257) = 1.001, *p* = .456 and 0.08 Hz: *F* < 1. Similar to Experiment 1, for the lower-performing participants reset accuracy was poor in the beginning of the session, but it improved during the session (see Fig. 12). On the other hand, for the higher-performing participants reset accuracy remained high throughout the session.

#### Time to first action (TFA)

**Fig. 13:**
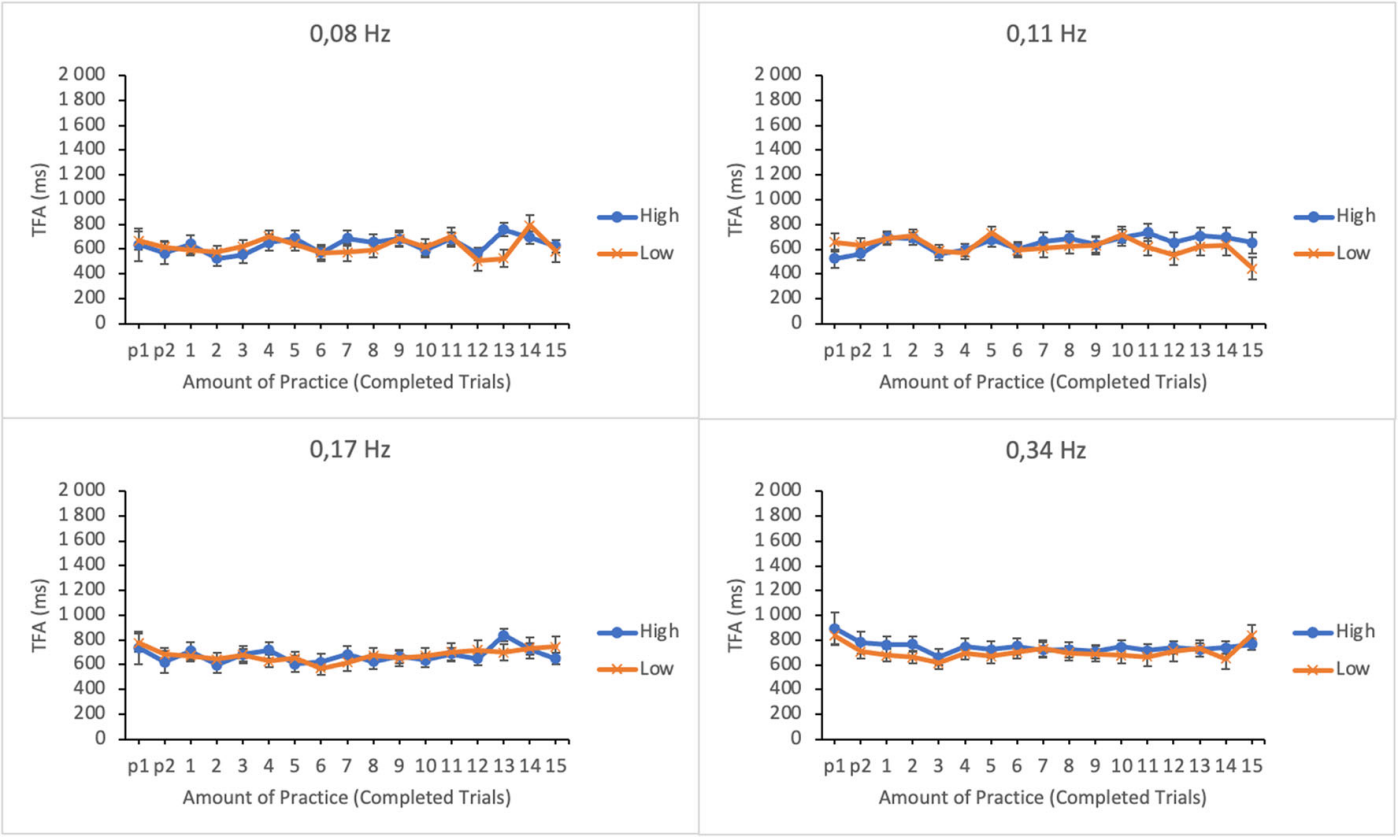
Estimated marginal means of time to first action in the subtasks as a function of amount practice and time-sharing performance of the lower (low-performing by the trial score) and the upper quartiles (high-performing by the trial score). The subtasks are presented in separate panes. Error bars represent the SEMs

The efficiency of time-sharing was analyzed by calculating the time to first action on each subtask. TFA data were submitted to a linear mixed effects analysis. Amount of practice, subtask event rate, and trial score as well as their interactions were entered as fixed effects in the model. Participants and its interactions with subtask event rate and amount of practice were entered as random effects. For illustrative purposes, the EMMs of TFA as well as their standard errors as a function of amount practice and trial score (the lower and the upper quartiles of trial score distribution) in the four subtasks are shown in Fig. 13.

As in Experiment 1 there was a significant main effect of subtask event rate on time to first action (see Table 2). The TFAs were longer for the subtasks with higher event rates. However, contrary to Experiment 1, the main effect of practice was not significant. The main effect of trial score was not significant. No significant interactions were found either.

### Discussion

In Experiment 2, we changed the subtask priorities continuously from trial to trial to see how participants adapt to the most difficult time-sharing situation, that is, to constantly changing subtask priorities. To our surprise, despite the high demands the task should have set to the attentional control system, the results were similar to those of Experiment 1. All participants adapted to the varying priorities of the subtasks almost instantly in terms of attention allocation. Here too participants’ behavior with the subtask of highest priority differed from the other subtasks. Higher performers invested a high percentage of trial time to the highest priority subtask from the start, whereas lower performers adjusted their attention allocation gradually towards higher levels. As in Experiment 1, nearly half of the participants also reported that they consciously focused their efforts on this subtask.

Visual sampling rate deviated from optimal in similar fashion as in Experiment 1. The most important subtask was undersampled while the other subtasks were sampled too frequently. This time no optimization on the subtask of highest event rate was observed. It is possible that the more complex task involving constant re-prioritization left participants no resources to adjust their performance further in this regard. As in Experiment 1, the prioritization of the subtasks was reflected in attention shifting. Times to first action were longer for the subtasks of higher importance, likely reflecting participants’ intent to secure successful resets.

In Experiment 1, longer dwell durations in the subtask of highest event rate during the first trials were associated with higher level of time-sharing performance. In Experiment 2, hints of similar but subdued behavior were registered. It suggests that here too more efficient performers recognized the need to observe this particular subtask more carefully, but the higher overall demands of the changing condition impeded their efforts.

In Experiment 2, participants’ behavior contradicts the prediction drawn from the assumptions of threaded cognition. A gradual optimization proposed by threaded cognition should have yielded highly inappropriate attention allocation in a time-sharing situation where subtask priorities change rapidly. However, a quick priority-based adaptation was observed instead.

## General discussion

The goal of this research was to investigate subtask prioritization, a key factor in successful time-sharing performance. A new experimental task was devised to enable the manipulation of subtask prioritization. The devised task generated resource conflicts; to solve them participants had to focus on the most important subtasks (operationalized as tasks requiring attention most frequently). Participants’ attention allocation was investigated by recording eye movements. In Experiment 1, we studied adaptation to a time-sharing environment in which priority order of the subtasks was kept constant from trial to trial. We found that participants were able to allocate their attention following the subtask priorities immediately from the very beginning. Participants who performed more efficiently in the overall time-sharing allocated their attention more accurately to the most important subtask than those who performed less efficiently.

In Experiment 2, we put participants’ attention control system to an ultimate resource conflict test. By changing the subtask priorities from trial to trial, we devised a highly demanding time-sharing environment resembling some real-life and professional situations, in which fast and agile adaptation is continuously needed to maintain sufficient level of performance. To our surprise, instead of exhibiting uncoordinated and disrupted attention allocation, participants adapted to subtasks’ requirements instantly in a highly controlled manner similar to Experiment 1. Here too higher and lower performing participants differed in their allocation behavior on the most important subtask.

### Results explained by threaded cognition

Threaded cognition’s most fundamental proposition is that there is no need in time-sharing for executive control in resource allocation. Resource allocation is based on subtasks following relatively simple rules. Subtasks (or threads) determine their resource needs independently, demanding resources greedily whenever needed and releasing them politely immediately when the need is fulfilled. Subtasks don’t interfere with each other unless their resource needs are in conflict. Conflicting needs are resolved according to subtasks’ urgencies. In the simplest form, urgency means that the task with the longest time since been served is the most urgent one and may receive resources first. Tasks adjust their urgencies gradually according to the feedback they get from the correspondence of received resources and the goal of the task.

Our results are inconsistent with these assumptions and predictions of the threaded cognition’s approach. Contrary to them, participants’ adaptation was generally so quick that we shouldn’t even call it adaptation, but more like an immediate perception of subtask priorities. Being able to adapt almost instantly and appropriately despite continuous changes in task requirements strongly points to involvement of a tight executive of some kind in resource allocation. It seems that the architecture proposed by threaded cognition renders adaptation to a new time-sharing situation too reactive, slow, and inflexible.

Only lower-performing participants’ performance on the subtask of highest event rate in Experiment 1 resembles a gradual manner of adaptation predicted by threaded cognition. It may be possible that their performance relied to some extent on a feedback-based optimization mechanism. It may be possible that in case subtask priorities are not quickly perceived and realized, adaptation may also happen more slowly through trial and error. However, the fact that lower performers also gradually improved their attention allocation in the changing conditions of Experiment 2 suggests that adaptation was not guided solely by this simple mechanism.

The observed differences in dwell times between the subtasks suggest that there may have been differences in the way participants processed information extracted from different subtasks. Longer dwells on high event rate tasks may reflect the perceived importance or priority of the task, which in turn denotes that participants were employing an allocation strategy involving prioritization. This is against the assumption of threaded cognition that no prioritization takes place in time-sharing. The fact that longer dwells were registered along with a high level of time-sharing in Experiment 1 may indicate that higher performers analyzed the most important subtask more intensively at first to create an accurate mental model of the required actions and also resisted premature attention shifts enabling them to absorb more information about the subtask status. In Experiment 2 such a difference in dwell durations was not observed, suggesting that no spare time was left even for the more efficient performers.

It should be noted that in their account of threaded cognition, Salvucci and Taatgen (2011) leave the door open for higher level control in time-sharing. They name the lack of metacognition as the most significant limitation of their theory. They define metacognition as an ability to reason about one’s own performance and adapt behavior accordingly. According to them, metacognitive processing is needed, for example, when adapting to distracting situations like those in traffic. Salvucci and Taatgen propose that metacognition acts as a separate thread within the framework of threaded cognition contending for resources like other task processes following the same rules. Salvucci and Taatgen mention that prioritization may be one of metacognition’s functions. Balanced, prioritized processing across threads can be realized as meta-cognitive threads that monitor other threads and direct them to increase or decrease processing based on external and internal demands. However, a more detailed description is missing about the functions of these metacognitive threads.

Clearly, to explain the results of the current study in terms of threaded cognition, a metacognitive thread of some kind is required. Apparently, the tasks we employed were complex enough to reach the boundaries of the simple resource allocation mechanism of threaded cognition. Probably the number of concurrent tasks was too high to be handled effectively by simple rules only. Presumably, continuous changes in the second experiment forced participants to employ higher level control in resource allocation.

### Results explained assuming executive control

The results for the high-performing participants are in line with the idea of effective executive control during time-sharing. Also, the fact that lower performing participants were able to improve their attention allocation and performance despite the continuous changes in Experiment 2 suggests that their prioritization was based on more abstract conceptual processing at least to some extent. The results suggest that participants had learned something from the previous trial’s setting that they could then use to help them to adapt to the next one. What they brought from one trial to the next might have been the comprehension of subtasks prioritization in a higher, more abstract conceptual level. If, for example, participants were able to realize how each subtask’s impact on the global task depends on its event rate and how event rate in turn is related to the position of the target bar, it could be possible to formulate a higher-level mental model that makes it easier and quicker to identify the changes between trials and improve performance in changing conditions. The ability to conceptualize the task into a mental model may be an essential differentiating factor between higher and lower time-sharing ability.

A related argument was put forth by Taatgen (2013). In an attempt to explain transfer of skills, he argues that procedural learning is too slow to reflect human performance in acquiring new skills, which can be almost instantaneous (see Anderson & Fincham, 1994, for the argument of slow procedural learning). In order to explain quick transfer and learning, he argues that cognitive control in the form of declarative knowledge has to be assumed in addition to procedural knowledge. This declarative control component (he calls them “operators”) represents and controls the order in which primitive procedural components are executed. This idea is consistent with the results of the present study.

What is more, once an effective attention allocation strategy is formulated, it has to be implemented during performance. Some people may be able to understand the situation and form a mental model, but not implement it. Implementing a certain fixed strategy during a hectic time-sharing situation where several subtasks are urging for response at the same time, would be a very difficult task for the attention control system. There would undoubtedly be a temptation to impulsively attend from time to time to subtasks of minor importance or other distractors. It is possible that besides the ability to quickly perceive priorities and formulate a strategy, the ability to resist distractions separates effective time-sharers from less effective ones. Effective time-sharers may possess a stronger endogenous control against random attentional fluctuations than less effective ones, whose control may in turn require more exogenous reinforcement to function effectively.

Clearly, problems exist with the theoretical approach assuming a tight executive. As argued above, the theoretical construct is too vague, it refers to a homunculus and makes computational modelling of human behavior difficult. However, Taatgen’s (2013) idea of using declarative memory for cognitive control may be one possibility to circumvent the homunculus problem.

## Conclusions

We found that the participants allocated their attention according to the subtask priorities. They sampled the most important subtasks more frequently, spent more time on them, and shifted their gaze earlier to them than to less important subtasks. Adaptation to the varying priorities of the subtasks was almost instant. Our study suggests that theories based on simple and liberal resource-allocation principles have serious problems in explaining human performance in demanding and dynamic resource conflict situations when immediate prioritization of attention is needed. The architecture proposed by a threaded cognition system makes adaptation to new and changing time-sharing situations too reactive, slow, and inflexible. Instead, avoiding accidents and incidents in real life clearly calls for a system capable of fast and authoritative control.
